# Comparison of Short-Term Outcomes after Robotic Surgery for Gastric Cancer in Elderly and Younger Patients: A Retrospective Cohort Study

**DOI:** 10.3390/cancers16162849

**Published:** 2024-08-15

**Authors:** Laura Fortuna, Fabio Staderini, Francesco Coratti, Fabio Cianchi

**Affiliations:** 1 Unit of Digestive Surgery, Careggi University Hospital, 50134 Florence, Italy; fabio.staderini@unifi.it (F.S.); corattif@gmail.com (F.C.); 2 Department of Experimental and Clinical Medicine, University of Florence, 50121 Florence, Italy

**Keywords:** gastric cancer, elderly patients, robotic surgery

## Abstract

**Simple Summary:**

There are still limited published data on the efficacy and safety of the robotic approach in the treatment of gastric cancer in elderly patients. The present study aimed at comparing the short clinical outcomes obtained in a group of elderly patients with those obtained in a group of younger patients after robotic surgery for gastric cancer. Although the elderly patients had more frequently comorbidities than the non-elderly patients, the incidence of serious postoperative complications, re-operation rate, 30-day mortality, and median hospital stay was similar within the two groups. Even the extent and adequacy of lymphadenectomy did not differ between the two groups. Our study suggests that robotic gastrectomy can be performed safely for elderly patients and may provide new insights into the validation of the robotic approach for treatment of gastric cancer in elderly patients.

**Abstract:**

Robot-assisted surgery has recently been introduced to overcome some drawbacks and technical limitations in performing laparoscopic gastrectomy. The aim of the present study was to evaluate the feasibility and safety of robotic gastrectomy in elderly patients. The study enrolled 143 patients who underwent robotic gastrectomy in a single high-volume centre. All patients were divided into two groups based on age: elderly group ≥ 75 years old (EG; n = 64) and non-elderly group < 75 years old (NEG; n = 79). Comorbidities were significantly more frequent in the EG (95.3%) than in the NEG (81%) (*p* = 0.011). Similarly, the percentage of ASA 3 patients was significantly higher in the EG than in the NEG (43.8% vs. 24.0%, respectively; *p* = 0.048). Nevertheless, the incidence of Clavien–Dindo grade III and IV complications did not differ significantly between the two groups (10.9% in the EG and 6.3% in the NEG; *p* = 0.852). Moreover, operative time, re-operation rate, mean number of harvested lymph nodes, 30-day mortality, and median hospital stay were similar within the two groups. Our study suggests that robotic gastrectomy can be performed safely for elderly patients. In particular, chronological age does not seem to affect either the clinical or oncological short-term outcomes after robotic gastrectomy.

## 1. Introduction

Gastric cancer is still the fifth most common cancer worldwide, with approximately 768,000 deaths per year according to the Globocan database [1]. In Italy, about 14,500 new cases of gastric cancer are estimated every year, with about 8700 deaths, representing 4% of all cancers in both sexes, the seventh in men, and the ninth in women [2]. Aging plays a major role in the development of this tumour and over the past decades the incidence has gradually increased in patients aged 65 and older [3]. Consequently, the increase in this population makes this cancer a major public health problem.

Radical gastrectomy is still the main method for the treatment of gastric cancer. However, elderly patients often suffer from multiple comorbidities and are usually considered as “fragile”. Frail patients are less able to tolerate surgical procedures, hospital stay, and immobilization stresses; as a consequence, surgery can be a major challenge for these patients, exhibiting an increase in postoperative morbidity and mortality, length of hospital stay, and intensive care unit admissions [4,5,6].

Minimally invasive surgery (MIS) for gastric cancer has been shown to be associated with less postoperative pain, quick mobilisation, better respiratory function recovery, reduced morbidity, and faster recovery, with similar oncologic outcomes compared to open surgery [7,8,9]. Previous studies on gastric cancer in the elderly demonstrated that laparoscopic assisted-gastrectomy can provide a shorter operative time, less intraoperative blood loss, and shorter length of hospital stay than open gastrectomy [10,11,12]. However, laparoscopic gastrectomy is considered a technically demanding procedure due to some difficult surgical steps such as lymph node dissection and anastomosis. Recently, robot-assisted surgery has been introduced to overcome some laparoscopic drawbacks and limitations; improved 3D vision, wristed instrument, tremor filtration system, and motion scaling can help surgeons perform the most challenging steps of gastric cancer surgery [13,14,15,16].

Currently, studies investigating the safety and efficacy of robotic gastrectomy for elderly patients are lacking [17,18,19]. The present study aimed to compare the short clinical outcomes obtained in a group of elderly patients with those obtained in a group of younger patients after robotic surgery for gastric cancer.

## 2. Patients and Methods

This is a retrospective cohort study concerning 143 consecutive patients who underwent robotic surgery for gastric cancer at the Digestive Surgery Unit (Careggi University Hospital) between January 2016 and March 2022. Data were retrieved from a prospectively maintained database. The exclusion criteria were as follows: (1) patients undergoing any gastric procedure for benign conditions; (2) patients with tumours located at the gastroesophageal junction; (3) patients with distant metastases or pre- or intra-operative T4 lesions (i.e., local invasion of other organs such as the spleen, pancreas, or peritoneum). We analysed patients’ characteristics such as gender, age, body mass index (BMI), American Society of Anesthesiologists (ASA) score, comorbidity, and previous abdominal surgery. Preoperative clinical staging was assessed in all patients by examining either a total body CT scan or endoscopic ultrasound when indicated. All patients were pre-operatively discussed in a multidisciplinary meeting involving surgeons, medical oncologists, radiologists, and pathologists. Those patients with clinical T3 and/or N+ tumours were scheduled for perioperative chemotherapy (usually fluorouracil–leucovorin–oxaliplatin–docetaxel regimen). The surgical performance was evaluated by a median operating time, conversion rate, and intraoperative complications. Morbidity and mortality were defined as postoperative complications and death within 30 days from surgery, respectively. Morbidity was categorized according to the Clavien–Dindo classification, and complications classified as ≥III were considered major [20]. Reoperation was defined as all surgical procedures that occur after primary surgery during hospitalization or within 30 days from the first intervention. Tumour characteristics were analysed, which included the grade of differentiation, depth of invasion (T-stage), and nodal status (N-stage). Tumour staging was assessed according to the eight TNM edition [21].

All patients were divided into two groups based on age: elderly group ≥ 75 years old (EG) and non-elderly group < 75 years old (NEG).

### Surgical Technique

**Figure 1:**
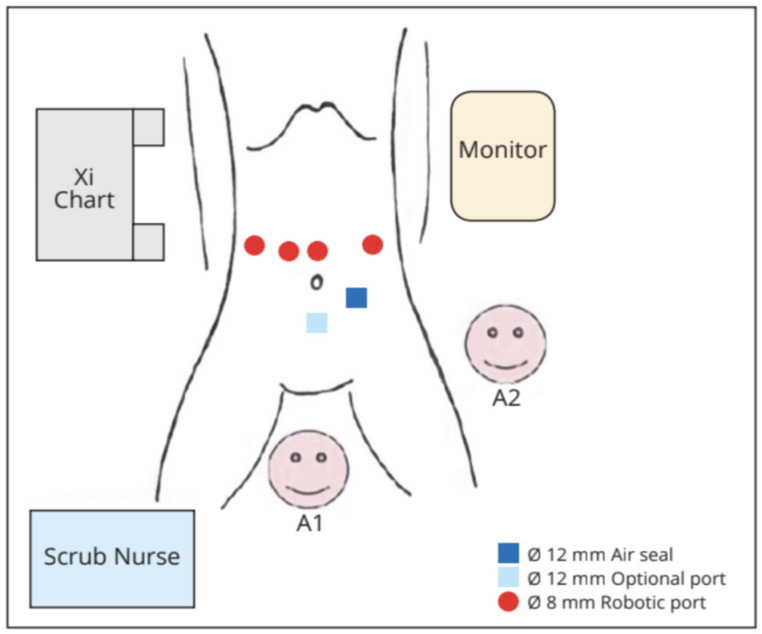
Operative room set-up. A1: table assistant number 1; A2: table assistant number 2.

**Figure 2:**
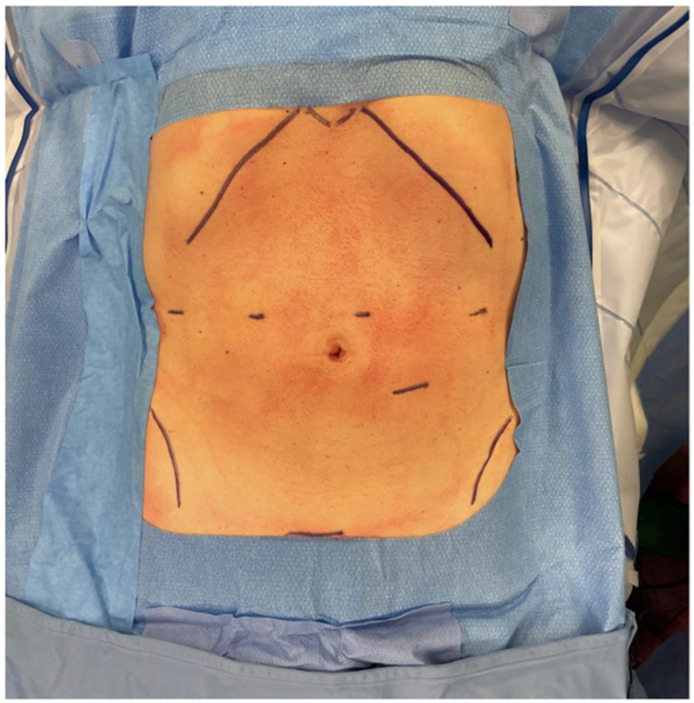
Position of the robotic trocars.

Under general anaesthesia, the patient was placed in a supine, reverse-Trendelenburg position with legs abducted. The robotic cart was positioned on the right side of the patient, at head level. We positioned the camera through the supraumbilical port. An active electrode or shear was held in the first robotic arm located on the left side of the patient. A fenestrated bipolar forceps and a ProGrasp forceps were held in the second and third arms, respectively, on the patient’s right side. The 12 mm trocar for the assistant was placed in the left sub-umbilical position. If needed by the assistant, an additional 5 mm could have been placed on the right transverse umbilical line (Figure 1 and Figure 2).

Subtotal gastrectomy: The operative procedure has been previously described [16]. Briefly, it involved the following steps: partial dissection of the left greater omentum and the lymph nodes along the left gastroepiploic vessels; dissection of the right omentum and the lymph nodes along the right gastroepiploic vessels; exposure of Henle’s trunk and division of the right gastroepiploic vein and artery for dissection of infrapyloric nodes; transection of the duodenum with either an endoscopic linear stapler or the robotic stapler just distal to the pylorus and reinforcement of the stump with a barbed running suture; division of the right gastric artery and dissection of the suprapyloric nodes and the nodes along the proper hepatic artery; dissection of the nodes along the common hepatic artery and the proximal splenic artery; division of the left gastric vein and artery and dissection of the nodes around these vessels and the celiac trunk; dissection of the lymph nodes along the lesser curvature and the right cardiac nodes; transection of the stomach on the upper third at least 5 cm above the tumour; mechanical intracorporeal gastro-jejunal anastomosis (either Billroth II or Roux-en-Y); and mechanical intracorporeal jejunal–jejunal anastomosis. The specimen was placed into an endobag and pulled out of the peritoneal cavity through the umbilical port, which was extended to a length of 4–6 cm.

Total gastrectomy: During total gastrectomy, the dissection of the left greater omentum was completed with the division of the short gastric vessel and the dissection of the relative lymph nodes. Then, the surgical steps were identical to those of the robotic distal gastrectomy except for the dissection of lymph nodes along the distal splenic artery and the dissection of the left cardiac lymph nodes. The distal esophagus was transected with a linear stapler, and a Roux-en-Y intracorporeal side to side esophago-jejunal anastomosis was performed. The jejunum–jejunal anastomosis was then performed either extra or intracorporeally.

Statistical analysis: Categorical variables within the EG and NEG were compared using Fisher’s exact test or the chi-square test. Quantitative variables were summarized by means and SEM or medians and range. The groups were compared using the Mann–Whitney test.

## 3. Results

Between January 2016 and March 2022, 143 patients were included in the study. There were 80 males (55.9%) and 63 females (44.1%). Among these, 64 patients were included in the EG and 79 in the NEG. The mean age was 66.2 (44–74) and 80.3 (75–90) years in the NEG and in the EG, respectively (*p* < 0.001). Comorbidities were significantly more frequent in the EG (95.3%) than in the NEG (81%) (*p* = 0.011). A total of 31 patients underwent perioperative chemotherapy (21.7%), 24 in the NEG (30.3%), and 7 in the EG (10.9%) (*p* = 0.007). As expected, the percentage of ASA 3 patients was significantly higher in the EG than in the NEG (43.8% vs. 24.0%, respectively; *p* = 0.048). The patient characteristics are shown in Table 1.

The mean operative time, defined as the time between the first incision to the closure of the last incision, was similar for the NEG (306.3 min) and the EG (284.5 min). A total of 129 patients had tumours located in the middle–distal third of the stomach, 71 (89.8%) in the NEG and 58 (90.6%) in the EG (*p* = 0.25). Only 14 patients (9.8%) suffered from proximal third gastric cancer, 8 in the NEG (10.1%) and 6 in the EG (9.4%). Consequently, distal gastrectomies were more frequent than total gastrectomies in both groups (72.2% vs. 27.8% in the NEG and 82.8% vs. 17.2% in the EG), and no significant difference was found between the two groups (*p* = 0.164). A significantly higher number of Billroth II reconstructions were performed in the EG than in the NEG (*p* = 0.007). No intraoperative complications occurred in either group. The incidence of Clavien–Dindo grade III and IV complications was higher in the EG (10.9%) than in NEG (6.3%), but the difference was not statistically significant (*p* = 0.852). Re-operation occurred in 2 patients within the NEG (2.5%) and in 4 patients within the EG (6.25%), but the difference was not statistically significant (*p* = 0.252).

There was no significant difference in the 30-day mortality (NEG: 0% vs. EG: 1.6%; *p* = 0.728) and the median hospital stay (NEG: 8 days vs. EG: 9 days; *p* = 0.131) between the two groups. Table 2 summarizes the operative outcomes.

The mean total number of harvested lymph nodes was similar in the two groups (NEG: 43.8 vs. EG: 44.7; *p* = 0.787). Although the percentage of stage I patients was higher in the NEG than in EG (44.3% vs. 26.5%, respectively), and the percentage of stage III patients was higher in EG than in the NEG (57.8% vs. 39.3%, respectively), the difference in the distribution of all patients, according to TNM stage, was not statically significant (*p* = 0.059). The oncological outcomes are shown in Table 3.

## 4. Discussion

There is still a limited amount of published data on the efficacy and safety of the robotic approach in the treatment of gastric cancer in elderly patients [17,18,19]. In the present study, we showed the results of one of the largest experiences of robotic gastrectomies in 64 patients older than 75 years. The EG was compared with the NEG, i.e., <75 years old, and as expected, a greater number of elderly patients presented with co-morbidities and higher ASA scores. Nevertheless, we found no significant difference in the incidence of Clavien–Dindo grade III and IV complications, 30-day mortality, and mean hospital stay between the two groups. In particular, there were no significant differences in the incidence of the two most harmful postoperative complications, i.e., anastomotic leakage and duodenal fistula. Our findings were similar to the results reported by the only previously published study addressing this issue: Okumura et al. [17] compared 49 patients older than 70 years with 321 patients younger than 70 years and found that chronological age does not affect recovery after robotic gastrectomy. These authors also showed that postoperative outcomes after robotic gastrectomy in the elderly were similar to those after laparoscopic gastrectomy in the elderly, despite a significantly higher operative time in the robotic group.

Due to the higher rates of cardiovascular and pulmonary comorbidities among elderly patients, longer operative times and prolonged exposure to pneumoperitoneum are considered the main problems during minimally invasive surgery in these patients. However, previous studies on laparoscopic gastrectomy in elderly patients have demonstrated that longer operative times had no impact on surgical outcomes [10,11,12]. Our findings seem to confirm these results since the incidence of postoperative complications did not differ between the EG and the NEG despite the fact that the mean operative times were longer than 4 h in both groups.

A recent meta-analysis [22] examined the surgical management of patients older than 80 years with gastric cancer and found that, in this group, operations were significantly faster and had lesser intraoperative blood loss compared to those in their younger counterparts. The authors explained these findings with the fact that D2 lymphadenectomy was less frequently performed in older patients, with the advantage of a lower risk of intraoperative bleeding but with the disadvantage of a lower number of harvested lymph nodes. Another meta-analysis dealing with the safety of gastrectomy in gastric cancer patients aged 80 or older [23] pointed out that octogenarian patients who underwent D2 had a higher risk of severe complications than younger patients, suggesting that standard surgery for gastric cancer should be limited in old patients. We performed D2 lymphadenectomy in both an NEG and EG, as demonstrated by the high number of lymph nodes harvested (more than 40 lymph nodes were examined in both groups), and observed a lack of significant difference in this number between the two groups (mean value 43.8 and 44.7, respectively). The finding that short-term clinical outcomes are similar in the groups suggests that robotic D2 lymphadenectomy can be safely performed in the elderly patients, thus providing a radical surgery even in this group of patients [24,25,26].

We found that the mean operative time in the EG was shorter than that in NEG even if the difference was not statistically significant. As previously analysed, this result was not due to a suboptimal lymphadenectomy but most likely to our choice to perform a Billroth II reconstruction in older patients rather than a Roux-en-Y reconstruction. Performing only one anastomosis permitted us to reduce both the operative time and the risk of anastomotic leakage without significantly affecting postoperative recovery.

The present study has some limitations. First of all, our study was conducted retrospectively, and the results were obtained from a single high-volume centre. Multicentre-randomized studies are necessary to definitively validate the robotic approach for gastric cancer treatment in the elderly. Additionally, we did not provide survival analysis and long-term oncological outcomes. This is an ongoing analysis but, in our opinion, it will be biased by the fact that a significantly lower number of elderly patients did not undergo perioperative chemotherapy based on a multidisciplinary decision.

## 5. Conclusions

In conclusion, our study suggests that robotic gastrectomy can be performed safely for elderly patients. In particular, chronological age does not seem to affect both clinical and oncological short-term outcomes after robotic gastrectomy.

## Figures and Tables

**Table 1:** Patient characteristics.

	Non-Elderly (n = 79)	Elderly (n = 64)	*p*
**Age** (years, mean ± SD)	66.2 (44–74)	80.3 (75–90)	<0.001
**Gender** (n, %) Female Male	47 (59.5) 32 (40.5)	33 (51.6) 31 (48.4)	0.398
**BMI** (mean ± SD)	23.3 (5.2)	24.0 (5.1)	0.409
**Comorbidities** (n, %)	64 (81.0)	61 (95.3)	0.011
**Perioperative chemotherapy** (n, %)	24 (30.3)	7 (10.9)	0.007
**ASA** (n, %) Class 1 Class 2 Class 3	11 (14.0) 49 (62.0) 19 (24.0)	6 (9.4) 30 (46.8) 28 (43.8)	0.048
**Previous abdominal surgery** (n, %)	12 (15.2)	13 (20.3)	0.508
**Tumour Location** (n, %) Distal third Middle third Proximal third	41 (51.9) 30 (38.0) 8 (10.1)	25 (39.0) 33 (51.6) 6 (9.4)	0.250

**Table 2:** Operative outcomes.

	Non-Elderly (n = 79)	Elderly (n = 64)	*p*
**Type of gastrectomy** (n, %) Subtotal Total	57 (72.2) 22 (27.8)	53 (82.8) 11 (17.2)	0.164
**Reconstruction** BII Roux-en-Y	45 (56.9) 34 (43.1)	51 (79.7) 13 (20.3)	0.007
**Operative time** (mean ± SD)	306.3 (72.6)	284.5 (79.8)	0.090
**Conversion** (n, %)	0 (0)	0 (0)	NA
**Complications** (n, %) Clavien–Dindo I–II grade Clavien–Dindo III–IV grade	13 (16.4) 5 (6.3)	18 (28.1) 7 (10.9)	0.852
**Re-operation** (n, %) Overall Bowel Obstruction Anastomotic Leakage Duodenal fistula	2 (2.5) 1 (1.2) 0 (0) 1 (1.2)	4 (6.25) 2 (3.1) 1 (1.5) 1 (1.5)	0.252
**Harvested nodes** (mean ± SD)	43.8	44.7	0.787
**30-day mortality** (n, %)	0 (0)	1 (1.6)	0.728
**Hospital stay** (days, median)	8 (7–10.5)	9 (7–11)	0.131

**Table 3:** Oncological outcomes.

	Non-Elderly (n = 79)	Elderly (n = 64)	*p*
**Grade of differentiation** (n, %) 1 2 3	13 (16.4) 37 (46.9) 29 (36.7)	5 (7.8) 39 (60.9) 20 (31.3)	0.160
**T classification** (n, %) is 0 1 2 3 4	2 (2.5) 2 (2.5) 17 (21.5) 18 (22.7) 37 (46.9) 3 (3.9)	0 (0.0) 1 (1.6) 11 (17.2) 8 (12.5) 38 (59.3) 6 (9.4)	0.355
**N classification** (n, %) 0 1 2 3	40 (50.7) 14 (17.7) 11 (13.9) 14 (17.7)	24 (37.5) 14 (21.9) 7 (10.9) 19 (29.7)	0.252
**TNM stage** (n, %) I II III	35 (44.3) 13 (16.5) 31 (39.2)	17 (26.6) 10 (15.6) 37 (57.8)	0.059
**Positive nodes** (mean ± SD)	2.9	4.4	0.111

## Data Availability

The data can be shared up on request.
